# The Principles and Practice of Obstetrics

**Published:** 1858-03

**Authors:** 


ART. V.— *The Principles and Practice of Obstetrics, including the Treatment of Chronic Inflammation of the Uterus, considered as a frequent cause of Abortion.* By HENRY MILLER, Professor of Obstetrical Medicine in the Medical Department of the University of Louisville. With illustrations on wood. Philadelphia: Blanchard & Lea. 1858. 8vo., p.p. 624.

Several years ago, Prof. Miller prepared a work entitled *A theoretical and practical treatise on Human Parturition.* This work, as the author remarks, was *printed,* but not in reality *published,* for falling into the hands of a book concern which soon became embarrassed, no efforts were made to give it circulation. The edition was nearly exhausted by the demand among those who had been pupils of the author, and beyond this circle the work was but little known. In other respects than the circumstances connected with its publication, it was eminently successful. It was prized by those who had listened with pleasure and profit to the oral instructions of Prof. Miller in the University of Louisville; and by critical readers both at home and abroad, it was highly esteemed. It was noticed in flattering terms in the reviews of this country and in Europe. We had the gratification, as a countryman and friend of the author, to find it occupying a conspicuous place in the library of the most distinguished professor of obstetrics in Great Britain, and to hear from his lips a warm eulogium of its merits. The friends of the author have long urged him to prepare a new edition. Laborious professional duties, and a mental temperament satisfied with the possession of knowledge and skill, without that craving for wide distinction which often stimulates to bibliographical labor, had occasioned delay in engaging in the task, till it began to seem doubtful whether it would ever be entered upon. We confess that we were taken somewhat by surprise by the announcement that the undertaking had been accomplished. Thorougly revised, much enlarged by the addition of new matter, and also improved by the introduction of illustrations, we have, at length, another edition under a new title. It is, in fact, essentially a new work, and is issued now by publishers who are abundantly able to do it justice.

We congratulate the author that the task is done. We congratulate him that he has given to the medical public a work which will secure for him a high and permanent position among the standard authorities on the principles and practice of obstetrics. Congratulations are not less due to the medical profession of this country, on the acquisition of a treatise embodying the results of the studies, reflections and experience of Prof. Miller. Few men, if any, in this country, are more competent than he to write on this department of medicine. Engaged for thirty-five years in an extended practice of obstetrics, for many years a teacher of this branch of instruction in one of the largest of our institutions, a diligent student as well as a careful observer, an original and independent thinker, wedded to no hobbies, ever ready to consider without prejudice new views, and to adopt innovations if they are really improvements, and withal a clear, agreeable writer, a practical treatise from his pen could not fail to possess great value. With this rare combination of the varied excellencies so desirable in an author of a work on a province of the *ars medicinæ* scarcely, if at all inferior in importance to either medicine proper or surgery, we need no other evidence that what he has written is deserving of diligent study on the part of the medical reader.

The work commences with a succinct but sufficiently full description of the pelvis and the sexual organs of the female. A chapter is then devoted to the clinical exploration of the female sexual organs. The consideration of pregnancy follows. The author then takes up the subject of abortion, and, next, the flooding of advanced pregnancy and incipient parturition. The remaining ten chapters are occupied with the various topics connected with labor.

We shall not undertake to review the work. Candor, indeed, obliges us to say that we have not, as yet, given it that thorough perusal which it claims. We have, however, examined it sufficiently to say that it bears the impress of the mental characteristics of the author. His conclusions are formed after mature deliberation, weighing well his own experience and according due weight to the opinions of others; but when deliberately formed, he does not hesitate to give them utterance, without regard to the authorities from whom he differs. The reader of the work who has not the advantage of a personal acquaintance with the author, cannot but rise from its perusal with the conviction that he has been made acquainted with the views of an honest, plain-spoken writer, who, with much learning and tact, combines the rare gift of good common sense.

Some of the doctrines advanced in this work are in strong contrast with those generally entertained. An instance of this kind relates to the necessity of manual delivery in all cases of unavoidable hæmorrhage. We are tempted to quote his remarks on this point contained in the preface. "It is very well known that in the latter part of the last century, the doctrine was peremptorily inculcated by Dr. Rigby that, in all cases of unavoidable flooding, i. e., flooding produced by the implantation of the placenta over the os uteri, delivery by turning and bringing the child by the feet, so soon as it is practicable, is the sole resource of obstetricy, on the due performance of which the salvation of the patient depends. This doctrine has been generally received and acted upon until recently; but, at this time, there are plain indications of dissatisfaction with it, and milder methods of treatment have been proposed under particular circumstances. But I do not know that any writer has proposed the abnegation of the practice of Rigby, in all cases, and the substitution of less harsh and hazardous expediments. This I have ventured to do. I could, indeed, do no less, for I have never met with an instance of unavoidable flooding in which I deemed it imperatively necessary to deliver by turning. On the contrary, it has always appeared to me that to deliver by the feet, where the head of the child presents, is a high-handed measure, not only in flooding, but under any circumstances of parturition, and one which is much more likely to be productive of evil than good, either as to the mother or child. Accordingly, repugnance to this kind of delivery constitutes a leading and distinctive feature of this work, which may not be pleasing to those who find themselves on their dexterity in operative manœuvres, but which, nevertheless, I am persuaded is its highest recommendation."

The author has entered pretty fully into the consideration of inflammatory affections of the uterus and displacements of that organ. He offers as an apology for introducing these subjects in a treatise devoted mainly to obstetrics, his desire to embody in a permanent form the results of his observations on these important classes of the sexual maladies of females, inasmuch as he does not contemplate the publication of a separate work on the diseases of females. It was quite gratuitous to apologise for what every reader who properly appreciates the importance of these affections, will receive with gratitude. We know that Prof. Miller's experience in these classes of maladies is large—perhaps as large, if not larger, than that of any practitioner in this country. We sincerely hope that the success of his present work may induce him to favor the profession with a treatise on the diseases of females. Without undervaluing the able works of Prof. Meigs on this subject, (and we yield to no one in our estimation of the abilities of this distinguished author,) there is abundant scope in this direction for farther bibliographical labor. We could mention another eminent practitioner (with whom we have the pleasure to be associated,) whose excellent judgment and rich store of experience admirably fit him for the task of writing a work on female diseases, but we much fear that the unremitted importunities of his friends will prove ineffectual.

Returning to Prof. Miller's work, we have only to add that we hope most sincerely it will be in the hands of every reading and thinking practitioner of this country. *

